# A Rare Case of Progressive and Invasive Adult Fibrosarcoma: Retroperitoneal Tumour Involving the Duodenum

**DOI:** 10.7759/cureus.97532

**Published:** 2025-11-23

**Authors:** Rahul Khullar, Toufic Ata, Nikhil Gupta, Preethi Rai, Anbalagan Pillai, Sai Babu Jonnada, Mohammed Mohseen

**Affiliations:** 1 Gastrointestinal Surgery, Medeor 24x7 Hospital, Abu Dhabi, ARE; 2 General Surgery, Burjeel Medical City, Abu Dhabi, ARE; 3 Gastroenterology, LLH Hospital, Abu Dhabi, ARE; 4 Pathology, Burjeel Medical City, Abu Dhabi, ARE; 5 Orthopaedics, Medeor 24x7 Hospital, Abu Dhabi, ARE; 6 Medical Oncology, Medeor 24x7 Hospital, Abu Dhabi, ARE; 7 Anaesthesia, Medeor 24x7 Hospital, Abu Dhabi, ARE

**Keywords:** abdominal fibrosarcoma, adult fibrosarcoma, lump abdomen, retroperitoneal malignancy, retroperitoneal neurofibroma, retroperitoneal sarcoma, retroperitoneal sarcomas, retroperitoneal sarcoma surgery, retroperitoneal tumour, sarcoma

## Abstract

Retroperitoneal soft tissue sarcomas are rare tumors encountered in clinical practice. Their presentation often involves enormous size, and they are located in close proximity to vital anatomical structures, which, along with preoperative diagnostic uncertainty, pose significant challenges for treating physicians. Cross-sectional imaging is essential for defining the characteristics of these lesions and for planning an appropriate management strategy. Preoperative imaging-guided histopathology is recommended for pathologically classifying these tumors before embarking on any treatment plan. Management of these cases varies with different histological subtypes. Surgical resection forms the mainstay of therapy. Neoadjuvant/adjuvant chemotherapy and radiotherapy may play a role in selected cases. Here, we present a progressive and invasive case of adult fibrosarcoma in a young male who underwent surgical resection for this tumor.

## Introduction

Retroperitoneal sarcomas are an uncommon and biologically heterogenous group of tumors arising from mesenchymal cells. They account for 10-15% of all soft tissue tumors [1]. More than 100 histopathological subtypes have been described, but the most frequent subtypes in order of frequency are well-differentiated liposarcoma (WDLPS)/dedifferentiated liposarcoma (DDLPS), leiomyosarcoma (LMS), solitary fibrous tumor (SFT), and malignant peripheral nerve sheath tumor (MPNST) [2]. Primary retroperitoneal neoplasms account for only 0.1-0.2% of all malignancies [3]. Among these retroperitoneal tumors, spindle cell neoplasms are very rare [4]. These tumors can arise in soft tissues, bones, and other parts of the body [5]. Adult fibrosarcoma is a highly aggressive soft tissue sarcoma. Mostly, they are located in either deep soft tissue or adjacent to bone. Rarely have they been reported in the retroperitoneum. As the retroperitoneum is a large, loose space, these tumors characteristically grow to a large size and most commonly present as vague abdominal pain or abdominal swelling. Treatment options should include anatomical characteristics and histological subtype [6]. Management of these cases is challenging due to the large size of the tumor, close proximity to vital structures, and low incidence in available literature. Here we present a case of a young male presenting with a visible large swelling in the abdomen with no other symptoms.

## Case presentation

**Figure 1:**
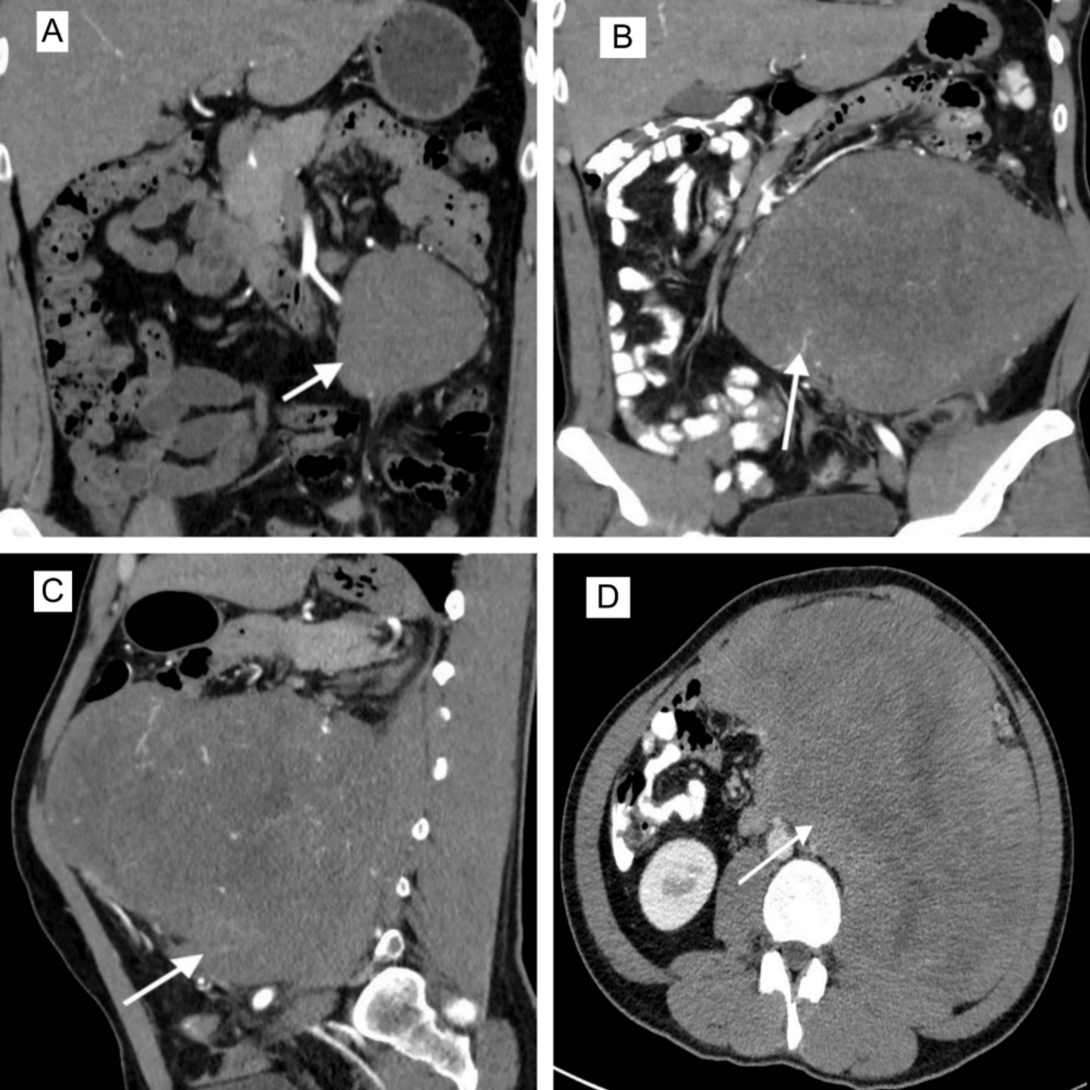
CT scan image of abdomen 1A (Coronal view)—Arrow showing retroperitoneal tumor on initial presentation; 1B (Coronal view), 1C (Sagittal view), 1D (Axial view)—Arrows showing large retroperitoneal tumor after second presentation

A 33-year-old male presented in an outpatient clinic with a feeling of a lump in the left lower abdomen. No abdominal pain, weight loss, anorexia, fever, or altered bowel habits. The patient had a history of an exploratory laparotomy in the past due to blunt abdominal trauma and intra-abdominal bleeding. No intestinal resection was done at that time. Vitals were within normal range. Per abdomen examination-No visible swelling, vague lump palpable (approximately 8 x 8 cm) in the left lower quadrant of the abdomen, not mobile. No tenderness over the lump. Computed tomography (CT) of the abdomen (Figure 1A) was done, which revealed a large, fairly well-circumscribed soft tissue density lesion (8.8 x 7.4 x 8.7 cm) with lobulated margins noted in the mesentery in the left lumbar region. It is solid and homogenous and shows a mild to moderate contrast study. No evidence of any necrosis or calcification, or fluid within. It is causing a pressure effect on the adjacent psoas muscle. The lesion is seen anterior to the left kidney and is causing displacement of the adjacent bowel loops. Multiple tortuous vessels are seen entering the lesion and traversing through it. Left kidney atrophic. Based on palpable swelling and a CT scan, the patient was advised to have further work done in the form of tumor markers, a biopsy of the lump, a tumor board discussion, and surgical resection eventually. Even after counseling the patient regarding the possibility of a tumor and its potential resectable status, he chose not to undergo further workup and treatment. He did not respond to any communication from the hospital.

One year later, the patient came into the outpatient clinic with a huge lump in the abdomen, a high-grade fever, a cough with sputum production, mild abdominal pain, and an aching nature. Weight loss of 2 kg occurred in the last 3 months along with intermittent constipation. Per abdomen examination, there is visible swelling in the left abdomen occupying almost the whole abdomen, predominantly on the left side. A palpable 20 x 15 cm lump occupying the left upper, left lower, and central quadrants in the abdomen, firm in consistency, immobile, and not moving with respiration. Vitals were within normal range. The patient was admitted due to a high-grade fever. The patient was diagnosed with influenza on a throat swab sample and was treated conservatively. No significant laboratory abnormalities.

CT abdomen (Figure 1B-1D) revealed a huge irregular heterogeneous soft tissue mass in the left mid-abdomen measuring approximately 24 x 17 x 16 cm in size. The mass appears to be primary retroperitoneal, arising from the left infrarenal region, and is extending anteriorly and laterally to abut the anterior and lateral abdominal wall. It is crossing the midline and reaching towards the right side, and displacing the aorta and IVC towards the right. The descending colon is compressed by the mass. The mass is closely abutting the left psoas muscle and compressing it. The margins of the mass are relatively well defined with no evidence of any direct invasion of any of the adjoining structures. Moderate heterogeneous enhancement of the mass is seen on post-contrast scans with central hypo-enhancing necrotic areas. No internal calcifications are seen. Left kidney atrophic

The case was discussed in the tumor board meeting. Due to the large size of the tumor and the compression of the tumor on the superior mesenteric artery and superior mesenteric vein, a plan was made for a CT-guided biopsy of the tumor for diagnosis and formulating a management strategy. Biopsy revealed a spindle cell neoplasm with neural differentiation.

**Figure 2:**
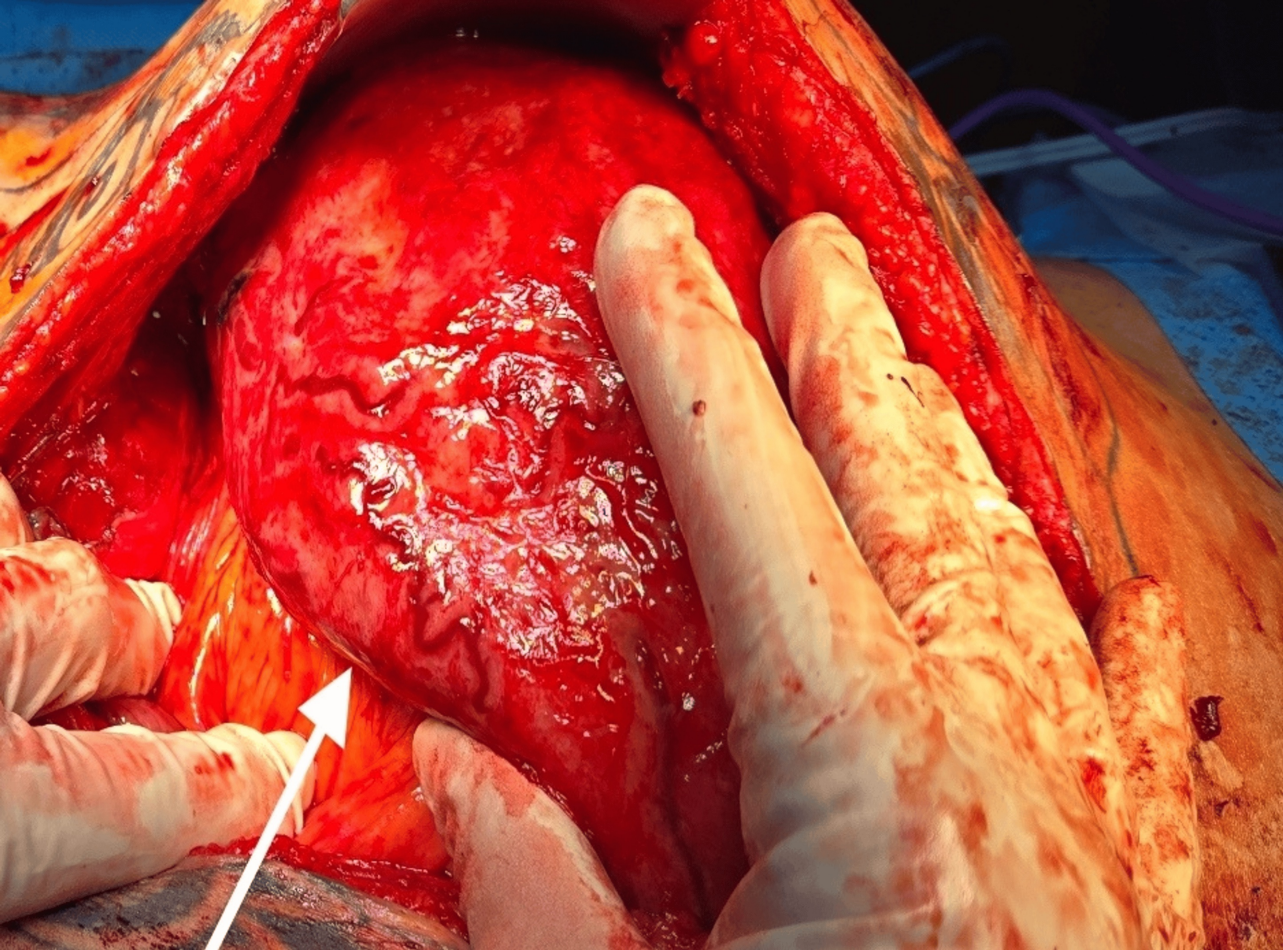
Intraoperative image of abdomen An arrow showing a tumour densely adherent to the small bowel mesentery.

**Figure 3:**
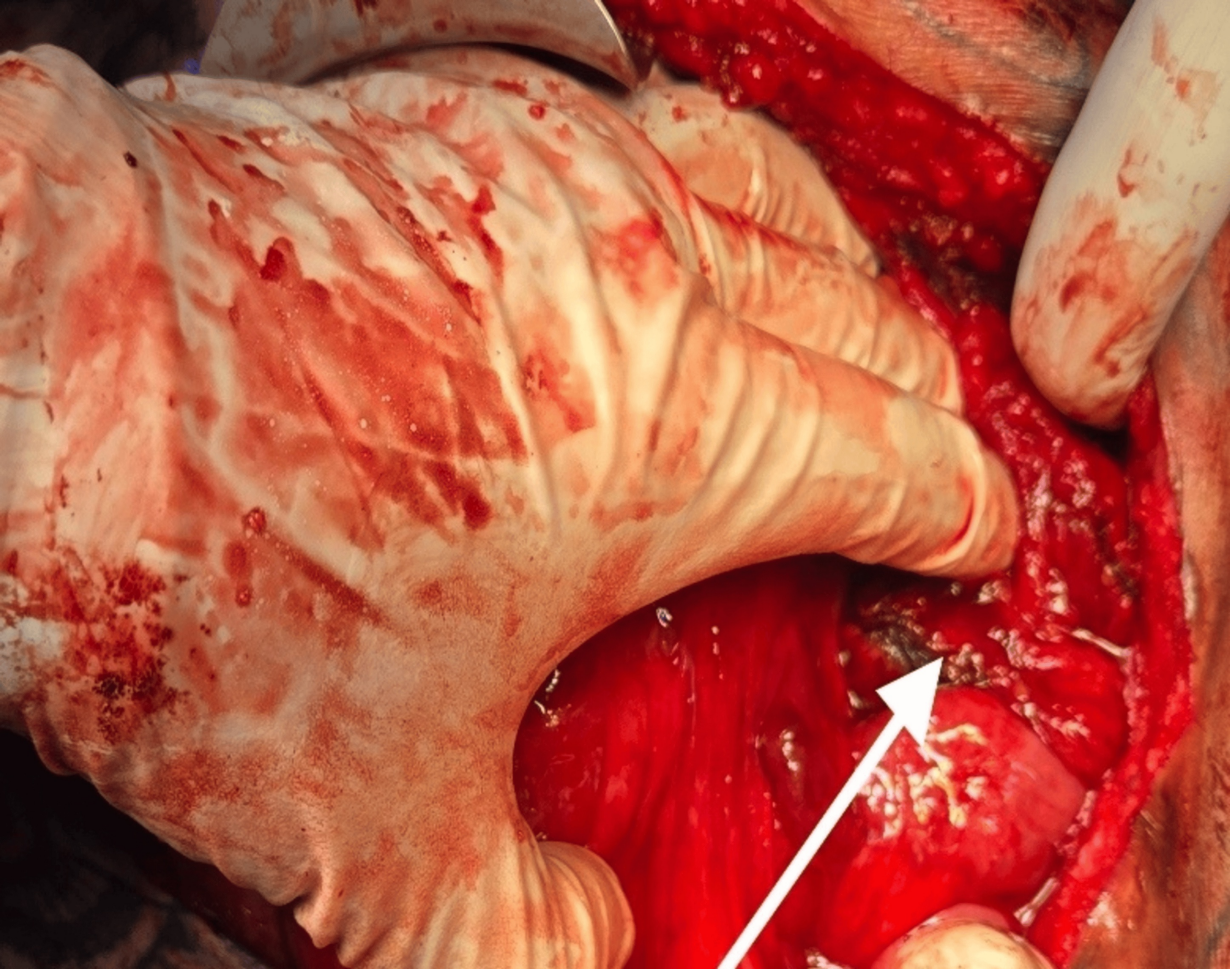
Intraoperative image of the pelvic region of the abdomen An arrow showing a tumour adherent to the intestinal and colonic loops in the pelvis.

**Figure 4:**
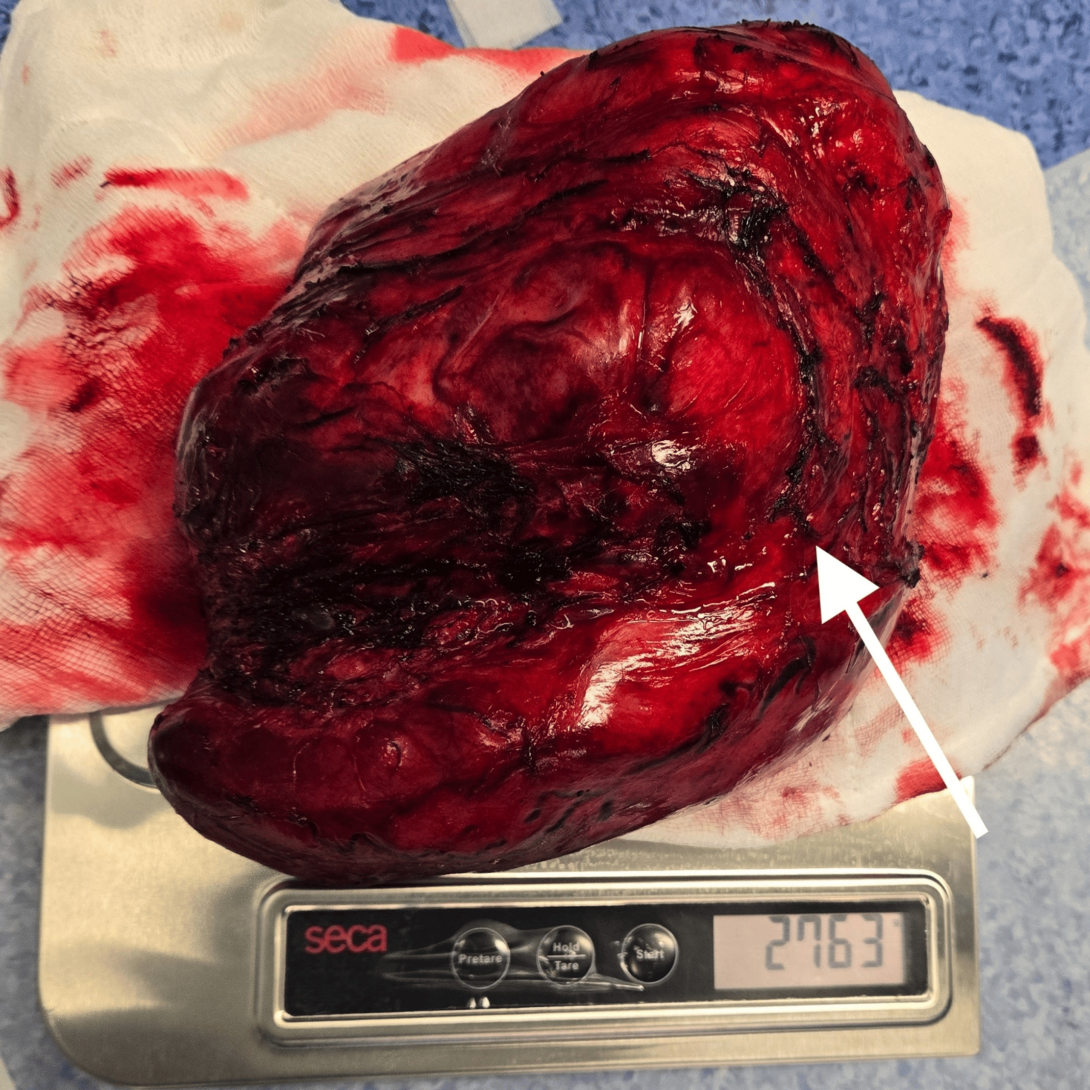
Post-operative image of the resected specimen Arrow showing resected tumour mass measuring 2.76 Kg.

As the tumor was surgically resectable on imaging, a plan for surgical resection was made after a repeat tumor board and multidisciplinary meeting. The patient had a good nutritional status with an Eastern Cooperative Oncology Group (ECOG) performance status of zero. Besides the atrophic left kidney, no comorbidities. The patient was taken for an exploratory laparotomy and resection of the tumor. Intraoperative Findings (Figure 2-3), large retroperitoneal tumor involving the central, left upper, and left lower abdomen (approximately 30 x 25 cm in size); densely adherent jejunal mesentery, sigmoid colon mesentery, duodenum (D4 part) just proximal to the duodeno-jejunal junction, Gerota's fascia, and psoas muscle (most likely site of origin). A tumor compressing on the superior mesenteric artery, aorta, and IVC, not infiltrating major vessels. Highly vascular tumor. Exploratory laparotomy with resection of retroperitoneal sarcoma with wide resection of the involved left psoas muscle, with sleeve resection of the involved duodenum with primary repair of the duodenum, with resection of part of the mesentery of the jejunum and sigmoid colon, with feeding jejunostomy. The descending colon and jejunum were not resected, as the blood supply was not compromised due to the uncompromised marginal vessels after detaching mesenteric attachments from the tumor. The possible origin of the tumor was from the left psoas muscle (R0 resection). The resected specimen weighed 2.76 kg (Figure 4). Surgical duration was 7 hours, and blood loss was 300 ml.

Postoperatively, the patient was managed in intensive care for two days. Postoperative day three: Feeding jejunostomy feeds were started after giving an initial trial feed, which the patient tolerated well.

On postoperative day five, the patient was started on oral liquids, and gradually the diet was increased. They passed flatus and feces on postoperative day five. On day six, the drain amount was 50 ml (serous), so the drain was removed, and the patient was discharged in a stable state.

**Figure 5:**
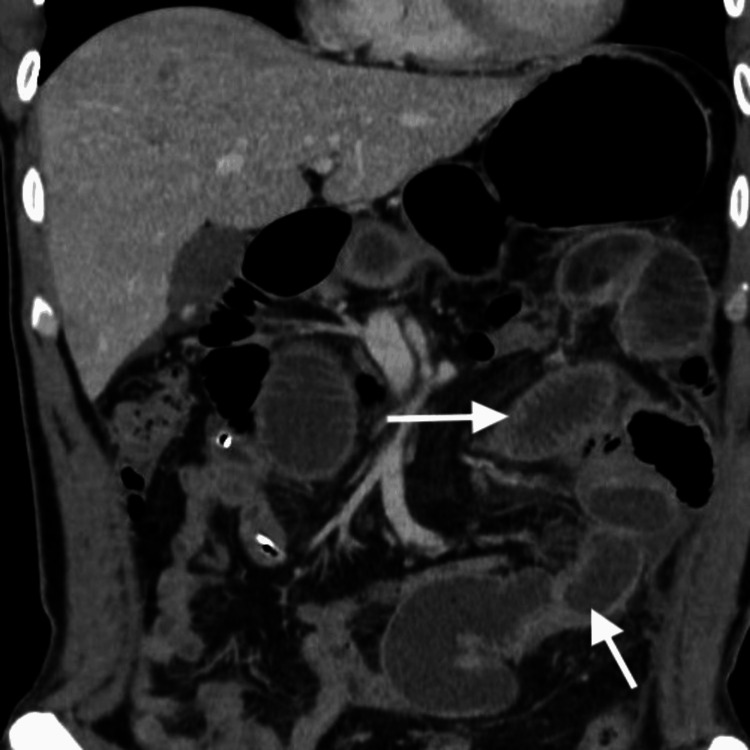
CT scan image of abdomen (Coronal view) Arrow showing postoperative adhesive bowel obstruction with small bowel thickening

After five days of discharge, the patient presented in the emergency room with complaints of abdominal pain, vomiting, inability to tolerate oral intake, and constipation. He was admitted. The CT abdomen (figure 5) showed mildly dilated duodenum and jejunum with multiple air-fluid levels. The wall of the bowel looks normal in thickness with normal enhancement on post-contrast images. Ileal loops look normal and nondilated. The patient was kept nil per oral, and conservative medical management was done. Total parenteral nutrition was given for three days. The patient passed flatus with no vomiting. Per abdomen was soft, no distention. The patient was given an oral diet, which he tolerated well, and gradually the diet was increased, and he was discharged after five days of admission. The feeding jejunostomy was removed six weeks after surgery. After six months of follow-up, the patient has no abdominal symptoms.

**Figure 6:**
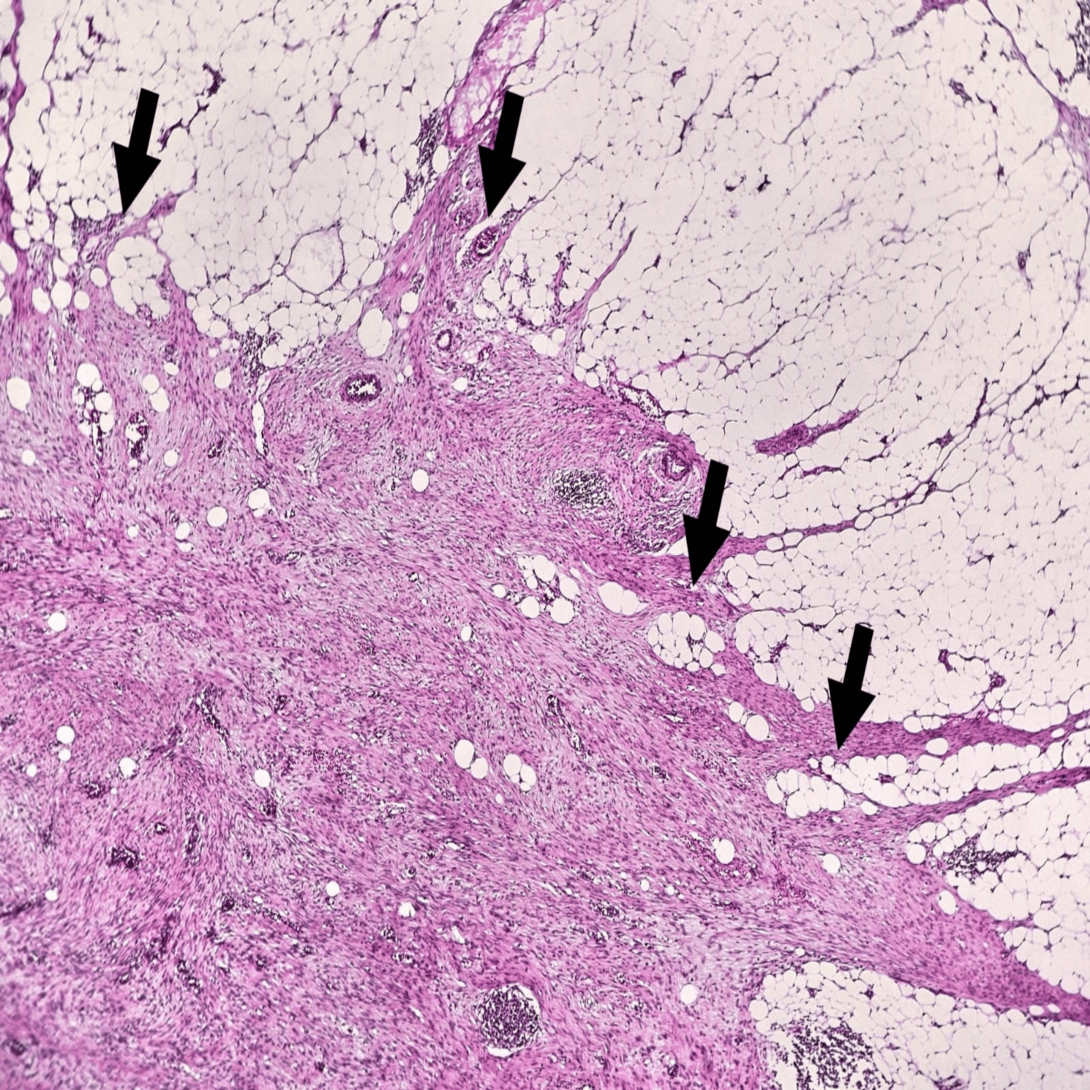
Hematoxylin and eosin stain Tumour border (arrows) showing infiltration of surrounding adipose tissue

**Figure 7:**
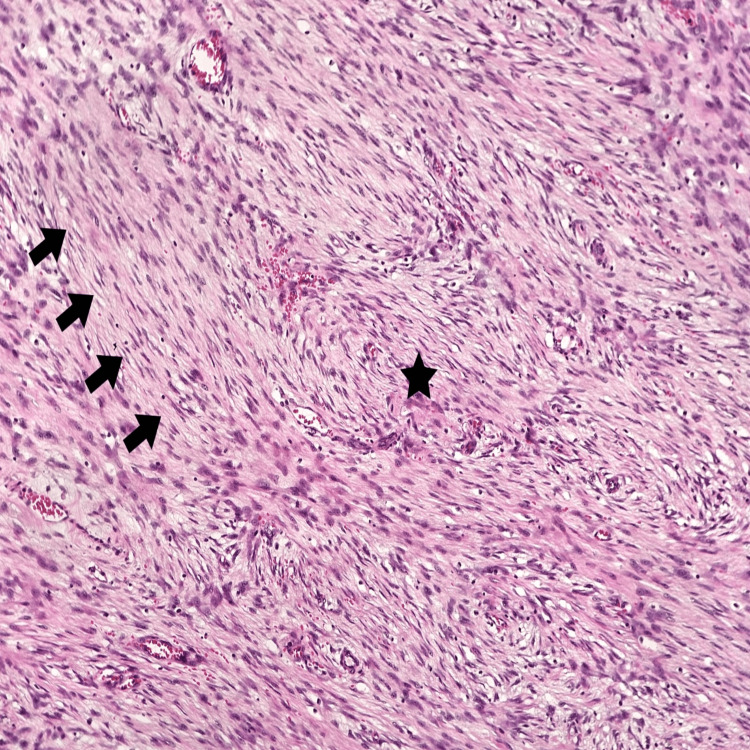
Hematoxylin and eosin stain Relatively monomorphic spindle cells, arranged in long, sweeping fascicles (arrows) with focal storiform areas (star).

**Figure 8:**
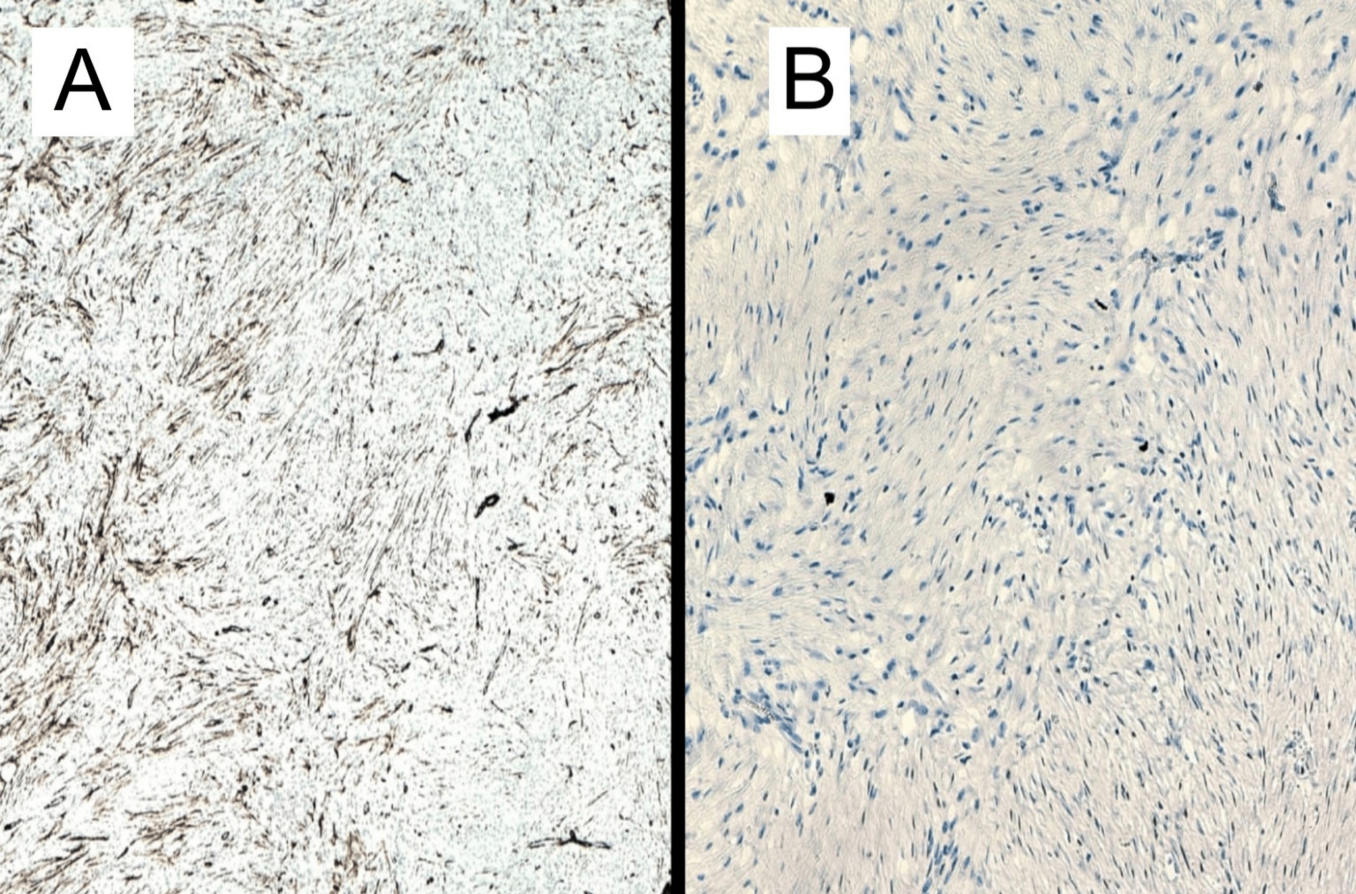
Immunohistochemistry (IHC) image Figure 8A—Tumor cells show limited expression of Smooth Muscle Actin (SMA); Figure 8B—Tumor cells show negative expression for Desmin, representing focal myofibroblastic differentiation.

Histopathology of the tumor suggested features are of a spindle cell neoplasm of uncertain differentiation, with low-grade morphological features (Figures 6-7). Immunohistochemistry (IHC) study with markers for gastrointestinal stromal tumors, smooth muscle tumors, peripheral nerve sheath tumors, adipocytic tumors, vascular tumors, skeletal muscle tumors, sarcomatoid carcinomas, inflammatory myofibroblastic tumors, and solitary fibrous tumors is inconclusive and did not reveal any definitive evidence of differentiation towards the corresponding lineages. Overall morphology and available immunohistochemistry findings favor a fibroblastic/myofibroblastic neoplasm. On IHC (Figure 8), the tumor cells are immunoreactive for SMA (patchy variable), CD10 (patchy variable), D240, S100 (5-10% of cells), and SOX10 (5-10% of cells). The Ki67 proliferation index is less than 5%. The tumor cells are immuno-negative for DESMIN, MYO-D1, CD117, DOG1, CD34, CD31, ALK1, CK-AE1/AE3, CK5/6, P63, ER, PR, STAT6, MDM2, BCL2, MUC4, CALRETININ, CYCIN-D1, and BETA-CATENIN (negative for nuclear staining). All controls show appropriate reactivity. No reportable gene fusions of known clinical significance or actionability were identified in the context of the underlying diagnosis. Negative for translocations involving USP6, NTRK, EWSR1, and FUS genes. Finally, a diagnosis of adult fibrosarcoma (a diagnosis of exclusion) was favored.

## Discussion

Retroperitoneal tumors are frequently diagnosed after incidental findings on a CT scan done for unrelated abdominal symptoms. If symptomatic, common complaints are nonspecific abdominal pain, back pain, abdominal swelling, and symptoms from compression of other organs (intestinal obstruction, urinary or gynecological symptoms). These are rare tumors, and surgical resection is the cornerstone of treatment [6]. CT scan with intravenous (IV) iodinated contrast is the imaging of choice. A CT scan is primarily useful for assessing areas of enhancement, necrotic areas, and tumoral extension in adjacent organs and for detecting metastasis and is also useful for guided biopsy with greater accuracy [7]. Findings of large, well-circumscribed, solid, vascular tumors, particularly with prominent feeding vessels, should alert the radiologist to a possible diagnosis of solitary fibrous tumor [7], as this was in our case. MRI of the pelvis can also be done to assess tumors with extensive pelvis involvement, though it was not done in our case. A CT scan provided sufficient information in our case.

An 18-Fluorodeoxyglucose-Positron Emission Tomography (18-FDG-PET) scan cannot differentiate low-grade tumors from benign lesions and has no routine role in diagnosis or assessment of tumor extension in retroperitoneal tumors [7]. In suspected retroperitoneal sarcomas, percutaneous image-guided core needle biopsy should be conducted before any treatment [8]. In our case, it showed a spindle cell neoplasm. Again, a tumor board discussion was done, and surgery was planned, but this process caused a delay in surgery of about 1 month, which may have resulted in involvement of the duodenum, not suspected on the CT abdomen. So delay in treatment should be avoided. If present, we advise another imaging before surgery to avoid intraoperative surprises. We did not do imaging in our case after these procedures and directly proceeded to surgery.

Preoperative nutrition status, comorbidities, ECOG performance status, and renal function tests should be assessed before surgical resection. The kidney is the most frequently resected organ in these cases. Our patient was a young, fit male with normal kidney function tests with a congenitally atrophic left kidney. Though from intraoperative experience, a normal left kidney would have made surgical resection more challenging than it already was.

Surgery is the mainstay of therapy for retroperitoneal tumors. Histology-guided surgery is advised according to the Spanish sarcoma research group guidelines [6]. In liposarcoma, local recurrence is more common and the most important factor in disease-related mortality [9]. Often, the approach of en bloc resection of involved organs is recommended. Left tumors should be resected en bloc with the left colon and left kidney, and in right-sided tumors, the right colon and right kidney should be excised with the tumor. Even the tail of the pancreas and spleen should be included in the resection if the tumor involves them or is in close abutment to the tumor [10]. Leiomyosarcoma usually originates from great blood vessels such as the inferior vena cava (IVC), renal, gonadal, or iliac veins and has a high incidence of distant metastasis and a low incidence of local recurrence. Adjacent organs should be preserved if not directly adherent to or invaded by the tumor. Solitary fibrous tumors have a low risk of recurrence and should be completely resected with negative margins.

Diagnosis of adult fibrosarcoma is almost always by exclusion using immunohistochemical and molecular techniques. They are characterized into 2 types: 1. Infantile/congenital type fibrosarcoma: It is of intermediate malignant potential and rarely metastasizes. 2. Adult Fibrosarcoma: A highly malignant tumor. Various markers, such as desmin, S100protein, CD31, CD34, epithelial membrane antigen (EMA), cytokeratin, and E-cadherin, are used to rule out other types of sarcomas. In fibrosarcoma, vimentin is often the only positively stained marker. Sometimes smooth muscle actin (SMA) can be detected as a sign of myofibroblastic differentiation. Ki-67, a cell cycle-associated nuclear antigen, is also used as a diagnostic marker for fibrosarcoma. Eighty percent of adult-type fibrosarcomas are found to be high-grade malignancies [11]. Regardless of grade, the overall five-year survival rate is 40-60% [11,12]

Surgery with R0 resection remains the standard of care in these highly aggressive sarcomas. Mostly, they are located in soft tissue or bones, so a 2 cm margin is also recommended. In retroperitoneal tumors closely abutting major vascular structures like the superior mesenteric artery and superior mesenteric vein, a margin of 2 cm is not always feasible. As these are rarely reported in the retroperitoneum, no defined line of management is available. In our case, part of the psoas muscle from which the tumor originated was removed en bloc with the tumor. In large high-grade tumors (> 5 cm in size) or high-grade features, radiation therapy is recommended. The role of chemotherapy is limited, as these tumors are not very responsive to chemotherapy [13].

In our patient, the feeding jejunostomy was removed six weeks after surgery, and they are currently under a six-month follow-up for any recurrence. Post-radiotherapy was not given in our case due to difficulty in irradiating the entire abdominal area with large doses of radiation.

## Conclusions

Retroperitoneal tumors present complex and challenging cases. Effective management requires a multidisciplinary team that includes a surgeon, pathologist, radiologist, and oncologist. Preoperative radiological and histopathological findings should inform the surgeons in selecting appropriate treatment options. Surgery for retroperitoneal tumors is frequently complex, necessitating effective intraoperative anesthesia management and careful postoperative critical care. Additionally, regular follow-up is essential to identify any recurrences quickly and manage them promptly.
